# Dynamic changes in serum carcinoembryonic antigen independently predict disease-free

**DOI:** 10.3389/fonc.2026.1896625

**Published:** 2026-10-14

**Authors:** Jing Xue, Qianqian Men, Xiaofei Tang, Xiaoyu Xie, Xiaoyan Li

**Affiliations:** 1 Department of Pharmacy, The Sixth Affiliated Hospital, Sun Yat-sen University, Guangzhou, China; 2 Guangdong Provincial Key Laboratory of Colorectal and Pelvic Floor Disease, The Sixth Affiliated Hospital, Sun Yat-sen University, Guangzhou, China; 3 Biomedical Innovation Center, The Sixth Affiliated Hospital, Sun Yat-sen University, Guangzhou, China; 4 Department of General Practice, The Sixth Affiliated Hospital, Sun Yat-Sen University, Guangzhou, China; 5 Department of Graceland Medical Center, The Sixth Affiliated Hospital, Sun Yat-Sen University, Guangzhou, China; 6 Guangdong Pharmaceutical University, Guangzhou, China; 7 Department of Oncology, The Sixth Affiliated Hospital, Sun Yat-sen University, Guangzhou, China

**Keywords:** CAPOX, CEA, CRC, dfs, efficacy

## Abstract

**Objective:**

This study aimed to evaluate the predictive value of serum carcinoembryonic antigen (CEA) changes for treatment response in colorectal cancer(CRC) patients receiving CAPOX chemotherapy, providing a reliable indicator for clinical efficacy monitoring.

**Methods:**

Clinical data of CRC patients who first received the CAPOX regimen at the Sixth Affiliated Hospital of Sun Yat-sen University between January 1, 2016 and January 31, 2026 were retrospectively collected. Parameters included operation date, chemotherapy administration dates, disease progression (PD) time, preoperative CEA, baseline CEA before chemotherapy, CEA before each cycle, and CEA within three months post-chemotherapy. Disease-free survival (DFS) was calculated. Patients were divided into Group 1: CEA is in the normal range (all values are ≤ 5ng/ml); Group 2: Decreasing or normalization type (not Group 1, and the last valid value ≤ 5ng/ml, or a decrease of ≥20% compared to the individual’s previous peak); Group 3: Persistent or new-onset abnormal type (the rest, that is, the last value > 5 and the decrease from the peak value < 20%). Survival curves were plotted using the Kaplan–Meier method, and multivariate Cox regression was used to assess correlations between CEA changes, treatment efficacy, and DFS.

**Results:**

Median DFS (mDFS) was 55.668 months (95% CI: 51.511–58.826) in Group 1 (n=84); 42.271 months (95% CI: 35.438–49.104) in Group 2 (n=47) and 19.980 months (95% CI: 11.198–28.762) in Group 3 (n=11).

**Conclusion:**

Dynamic changes in serum CEA have good predictive value for the efficacy of CAPOX chemotherapy in CRC. This predictive value is primarily significant in TNM Stage III patients and serve as an independent prognostic factor. Patients with decreased or persistently normal CEA levels have a better prognosis.

## 1 Introduction

The CAPOX regimen [capecitabine(CAP) combined with oxaliplatin(OXA)] is one of the commonly used first-line chemotherapy regimens for CRC, which has the advantages of convenient oral administration, definite efficacy, and relatively controllable toxic side effects. Although CAPOX reduces recurrence risk, clinical outcomes remain heterogeneous, and some patients still experience recurrence. Although the CAPOX regimen has definite efficacy, there are significant individual differences in efficacy among different patients, and long-term application may cause peripheral neuropathy, hand-foot syndrome and other toxic side effects. The overall prognosis of patients is generally poor (1).Therefore, it is necessary to seek simple and dynamic indicators to monitor the efficacy of chemotherapy. Currently, clinical practice mainly relies on periodic imaging examinations to assess the efficacy, but this assessment method has a lag, and it usually takes 2–3 chemotherapy cycles before a judgment can be made, making it difficult to identify drug-resistant patients at an early stage. Therefore, finding a simple, cost-effective, and dynamically monitorable efficacy prediction indicator to identify chemotherapy-sensitive patients at an early stage is of great significance for achieving individualized treatment for CRC.

CEA is the most classic and widely used serum tumor marker for CRC. CEA is an acidic glycoprotein with extremely low levels in normal adult serum. When the mucosal cells of the colon and rectum undergo malignant transformation, the synthesis and release of CEA significantly increase. From the perspective of pathophysiology, CEA not only reflects the tumor burden but also participates in the adhesion, invasion, and immune escape processes of tumor cells. Its dynamic changes can indirectly reflect the alteration of tumor biological behavior, which is related to chemotherapy resistance. The mechanism by which tumor cells develop resistance to the CAPOX regimen is complex and involves overexpression of drug efflux pumps, mutations in drug targets (such as thymidylate synthase), enhanced DNA damage repair ability, and the presence of tumor stem cells. When these mechanisms are activated, the DNA cross-linking damage induced by OXA cannot be effectively converted into cell death, and the active metabolite of CAP also fails to inhibit DNA synthesis, resulting in an increase in CEA levels instead of a decrease. A large number of studies have confirmed that the level of CEA is closely related to the tumor stage, recurrence risk and long-term survival of patients with CRC (2) (3) (4) (5). Moreover, some research has pointed out that the combination of serum CEA with other indicators can provide a strong basis for clinical diagnosis and treatment (6) (7) (8) (9). However, at present, the research on the dynamic changes of serum CEA during chemotherapy and its predictive value for the efficacy of the CAPOX regimen is not sufficient, especially lacking sufficient verification in the domestic population.

The innovation of this study lies in the following aspects: First, it focuses on the dynamic changes of CEA throughout the chemotherapy process, rather than a single absolute value, which can more accurately reflect the treatment response; Second, based on CEA, a simple and dynamic efficacy prediction model is constructed, providing an auxiliary decision-making tool for clinicians, which helps to promptly adjust the treatment plan and promote individualized treatment; Third, the efficacy assessment is associated with DFS, providing comprehensive prognostic information from “whether it is effective” to “how long the benefit lasts”.

This study retrospectively collected the clinical data of CRC patients who received the CAPOX chemotherapy regimen at the Sixth Affiliated Hospital of Sun Yat-sen University. The correlation between the changes in CEA levels and the efficacy of chemotherapy, as well as DFS, was analyzed, thereby providing a reliable basis for individualized treatment and efficacy monitoring of CRC patients.

## 2 Materials and methods

### 2.1 Research data

#### 2.1.1 Research subjects

This is a single-center, observational, retrospective study and analysis. 257 CRC patients admitted to the Sixth Affiliated Hospital of Sun Yat-sen University from January 1, 2016 to January 31, 2026 were selected as the research subjects. A total of 142 people were included in the study. The screening process is shown in **Figure 1**. Inclusion criteria: (1)Patients with CRC aged 18 years or older; (2) Patients with pathological diagnosis of TNM stage 2-3; (3) Complete CEA monitoring records (baseline and before each cycle) and imaging assessment data; (4) Patients’ diagnosis and treatment conformed to the “Chinese Guidelines for the Diagnosis and Treatment of CRC”; (5) Using CAPOX as a neoadjuvant and adjuvant treatment plan. Exclusion criteria: (1) Complicated with other types of malignant tumors; (2) With severe liver, kidney, or heart dysfunction; (3) Patients undergoing neoadjuvant and adjuvant radiotherapy; (4) Patients with TMN stage 0-1; (5) Missing key CEA or imaging data; (6) With infectious or immune system diseases; (7) Received other systemic anti-tumor treatments during chemotherapy. This study was approved by the Ethics Committee of the Sixth Affiliated Hospital of Sun Yat-sen University in Guangzhou, China(2026ZSLYEC-384).

**Figure 1:**
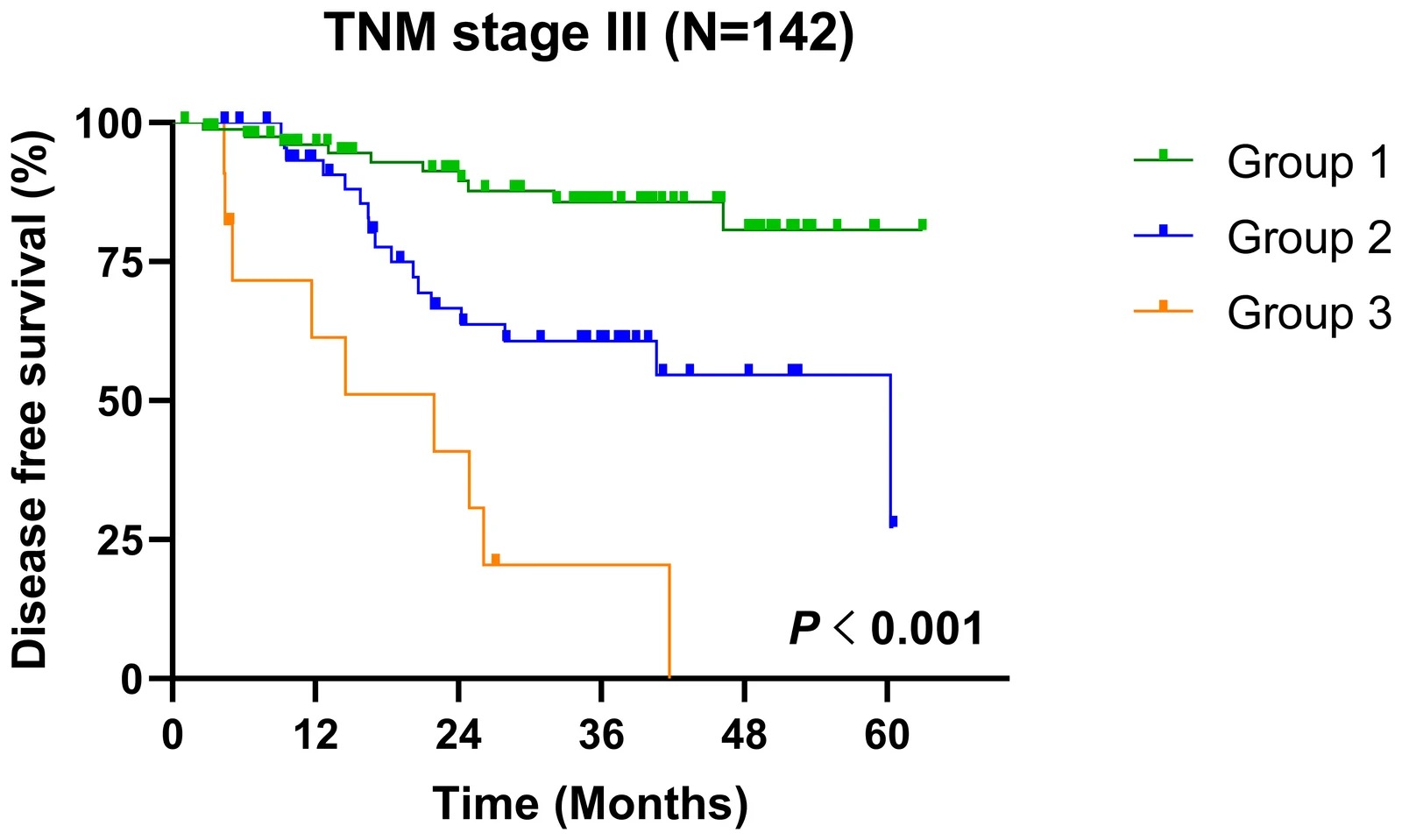
Patient screening flowchart.

#### 2.1.2 Data collection

Data was collected through the electronic medical record system of the Sixth Affiliated Hospital of Sun Yat-sen University. The clinical data of patients included gender, age, height, weight, primary site, TNM stage, operation time, first medication time, last medication time, PD time, and serum CEA level before each chemotherapy session.

#### 2.1.3 Treatment plan

The Capox regimen involves the combination of OXA and CAP. OXA is administered intravenously at a dose of 130mg/m^2^ for a duration of more than 2 hours on the first day; CAP is taken orally at a dose of 1000mg/m^2^ twice daily. A 21-day period constitutes one treatment cycle. A therapeutic efficacy assessment is conducted every 2 cycles.

#### 2.1.4 Sample size estimation

In the retrospective design of this study, based on the empirical rules of statistical modeling, the Cox regression model requires that each included independent variable should correspond to at least 10–15 outcome events. In the model of this study, the number of independent variables included was 5 (age, gender, TNM stage, primary tumor location, CEA change), and the number of events should be 50-75. The study ultimately included 257 patients, and clinical observations revealed that the incidence of progression/recurrence events was approximately 30% (approximately 77 cases), which met the basic requirements of the Cox regression analysis for the number of events.

### 2.2 Outcome definition

The primary outcome measure of this study is DFS, defined as the time from the first administration of the CAPOX chemotherapy regimen until the first occurrence of any of the following events: (1)radiologically confirmed disease recurrence or metastasis (in accordance with the RECIST 1.1 version); (2)a second primary tumor; (3)death due to any cause. For the patients in this cohort who received chemotherapy, the first radiologically confirmed PD is operationally equivalent to the recurrence or metastasis event. The assessment of disease progression is made according to the Response Evaluation Criteria in Solid Tumors (RECIST 1.1 version), and is evaluated by the radiology and oncology doctors. In case of disagreement, the oncology doctor makes the final decision. The normal reference range for serum CEA in our laboratory is 0 - 5.0 ng/mL, with a detection lower limit of 1.73 ng/mL.

### 2.3 Statistical methods

Statistical analysis was conducted using SPSS 21.0 software. For the measurement data that followed a normal distribution, they were expressed as mean ± SD and compared using t-test. For the measurement data that did not follow a normal distribution, they were represented as median(IQR) and compared using rank sum test. Count data were compared using χ2 test. The survival curve was plotted using the Kaplan-Meier method, and single-factor survival analysis was performed using Log-rank test. Report HR, 95% CI and bilateral P value; Estimate the median follow-up time using the reverse Kaplan-Meier method. A difference was considered statistically significant when P<0.05.

## 3 Results

### 3.1 Baseline characteristics of patients

From January 1, 2016 to January 31, 2026, a total of 257 patients met the study criteria and were included in this study. Among the 257 patients, 158 were male and 99 were female; the age ranged from 27 to 80 years, with a median age of 58 years. The 257 patients were divided into three groups based on the changes in CEA (Group 1: CEA is in the normal range (all values are ≤ 5ng/ml); Group 2: Decreasing or normalization type (not Group A, and the last valid value ≤ 5ng/ml, or a decrease of ≥20% compared to the individual’s previous peak); Group 3: Persistent or new-onset abnormal type (the rest, that is, the last value > 5 and the decrease from the peak value < 20%);. The basic information of the three groups of patients is shown in **Table 1**.

**Table 1:** Summary of case handling.

CEA group	Total number	Number of events	Censorship	
			N	Percentage
1	84	10	74	88.1%
2	47	17	30	63.8%
3	11	9	2	18.2%
Overall	142	36	106	74.6%

### 3.2 Survival data

The median DFS for group 1 was 55.668 months (95% CI: 51.511 - 59.826); the median DFS for group 2 was 42.271 months (95% CI: 35.438 - 49.104), with a mean of 60.270 months (95% CI: 22.530 - 98.010); the median DFS for group 3 was 19.980 months (95% CI: 11.198 - 28.762), with a mean of 21.970 months (95% CI: 6.585 - 37.355). See **Table 2** for details.The mDFS of Group 1 was 13 months longer than that of Group 2, and 35 months longer than that of Group 3. The DFS of Group 1 was significantly better than that of Group 2 and Group 3 (Log-rank P = 0.013), as shown in **Table 3**.The Kaplan-Meier survival curve for patients with TNM stage 2 showed no significant difference among the three groups (P = 0.101), as shown in **Figure 2**. The Kaplan-Meier survival curve for patients with TNM stage 3 indicated that there was a statistically significant difference in survival rates among the three groups (P < 0.001), as shown in **Figure 2**.

**Table 2:** The mean and median of the survival table.

CEA Group	^ą^Mean value				Mean value			
	Estimate	Standard error	95% confidence interval		Estimate	Standard error	95% confidence interval	
			Lower limit	Upper limit			Lower limit	Upper limit
1	55.668	2.121	51.511	59.826				
2	42.271	3.486	35.438	49.104	60.270	19.255	22.530	98.010
3	19.980	4.480	11.198	28.762	21.970	7.850	6.585	37.355
Overall	47.668	2.083	43.586	51.750	60.270	11.757	37.226	83.314

^ą^If the estimated value is censored, it will be restricted to the longest survival time.

**Table 3:** Overall comparison.

	Chi-square	df	Sig,
Log Rank(Mantel-Cox)	37.125	2	.000

**Figure 2:**
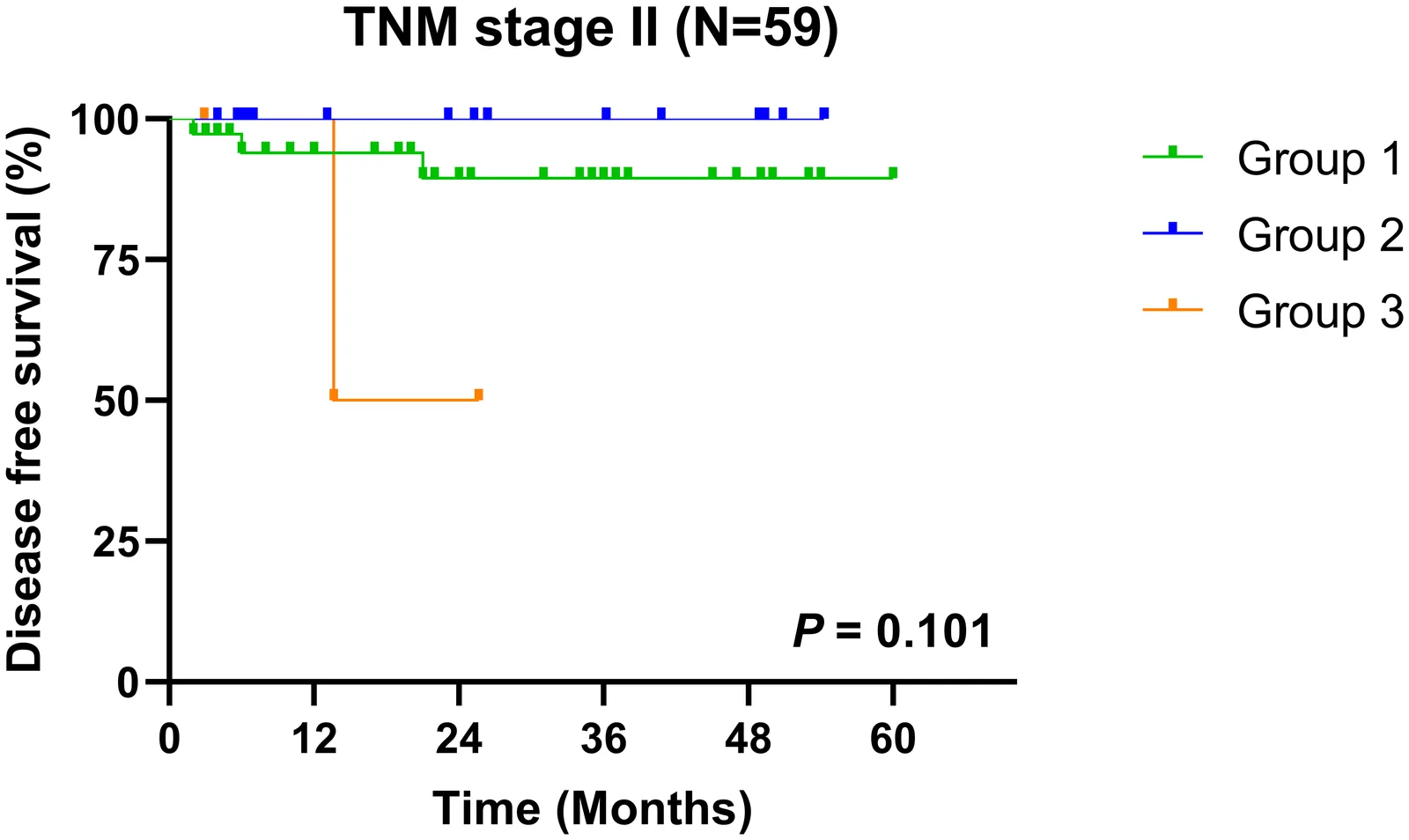
Kaplan-Meier survival curve.

### 3.3 Other notes

Our data were simultaneously included during the entry process in terms of “KRAS/NRAS/BRAF gene mutation status, MSI status, postoperative pathological risk factors, and surgical margin status”. However, since the auxiliary patients were not required to undergo genetic testing by default, there were many missing data, making subgroup analysis impossible.

## 4 Discussion

### 4.1 Clinical value of serum CEA

Studies have shown that changes in the serum levels of tumor markers typically occur earlier than the appearance of clinical symptoms. The detection of these biological markers is of great clinical significance for the early screening and prognosis monitoring of tumor diseases. The results of this study indicate that for CRC patients with TNM stage 3, the median progression-free survival of the patients in group 1 was significantly longer than that of the patients in groups 2 and 3, and the difference between the two groups was statistically significant.

CEA, as the most classic serum tumor marker for CRC, is also extensively involved in key biological processes such as regulation of the tumor immune microenvironment, immune escape, and angiogenesis (10).

Furthermore, numerous previous studies have confirmed the close relationship between serum CEA and the diagnosis and prognosis of CRC, and it is often detected in combination with other indicators. The combined use of serum CEA, ESM-1, and D-D tests is helpful for the clinical diagnosis of CRC (11) (12).Lerch et al. found that CRC at a later stage tended to have higher CEA levels, and thus the prognosis was often worse (13). Compared with previous studies, this study focused on the specific chemotherapy regimen of CAPOX. Previous studies mostly took the absolute value of CEA as the research object and then compared the changes in CEA before and after treatment. This study directly focused on the expression changes of CEA during chemotherapy (decrease, increase, always below the detection value) as the research object and grouped accordingly, which is conducive to reflecting the real response process of the tumor to chemotherapy.

### 4.2 Interpretation of abnormal results

The main conclusion of this study, which is that the dynamic change of CEA is an independent predictor of DFS in CRC patients receiving CAPOX chemotherapy. At the same time, this finding also suggests that in the regression analysis of retrospective studies, for subgroups with too small sample size or too few events, the parameter estimation results should be interpreted with caution. Furthermore, in this study, the TNM stage did not demonstrate independent predictive value for DFS in the multivariate Cox regression analysis (P > 0.05), which differs from the results of some previous studies. Multivariate Cox regression analysis revealed that TNM stage was an independent predictor of prognosis (14) (15). III-IV stage is an independent risk factor for poor prognosis in patients undergoing CRC surgery (16) (17) (18). The possible reasons for this difference include: (1)In this study, the sample size of patients in stage IV was relatively small (only 27 cases, accounting for 10.5%), which might have limited the ability to detect the prognostic value of TNM staging; (2)The population in this study was mainly composed of patients receiving adjuvant chemotherapy (accounting for 84.0%), and the intervention of the CAPOX regimen might have partially weakened the prognostic impact of TNM staging itself; (3)Different studies have different definitions of prognostic outcomes, which might also lead to differences in the results.

### 4.3 Circulating Tumor DNA (ctDNA) and CEA exert a complementary and synergistic effect on each other

In recent years, ctDNA monitoring has achieved remarkable results in CRC. However, its relationship with CEA is not a simple substitution but rather a complementary and synergistic one. The clinical value of ctDNA highly depends on the treatment stage and the monitoring purpose. This indicates that the positive result of ctDNA reflects “tumor residual signals”, but does not equate to “an immediate intervention indication”. Compared with CEA, ctDNA has better sensitivity (91.4% preoperatively vs 42.9%) and specificity for recurrence monitoring (0.95 vs 0.88) (19, 20). However, the IDEA-France analysis indicates that CEA remains an independent prognostic factor (21). The reason lies in the fact that the two monitor different temporal and spatial dimensions: ctDNA reflects the real-time proliferation kinetics, while CEA is affected by metabolic clearance and microenvironment remodeling, which is something that ctDNA cannot match. Therefore, our research group believes that based on different monitoring purposes, ctDNA and CEA can cooperate with each other. In the early efficacy warning, the early kinetics of ctDNA is the core. In the long-term efficacy decision-making, for example, introducing the bidirectional verification coefficient of ctDNA-CEA. If ctDNA is negative but CEA is elevated 4 weeks after surgery, chemotherapy should not be abandoned easily. If ctDNA is positive but CEA is normal and imaging is negative, it is recommended to re-examine within 4 weeks to confirm persistent positivity before intervening to avoid over-treatment. In summary, ctDNA is the “real-time microscope” and CEA is the “macroscopic barometer”. The combined dynamic assessment of the two is more conducive to the entire management of CRC.

## 5 Innovativeness

The innovation of this study lies in the following aspects: First, it focuses on the dynamic changes of CEA throughout the chemotherapy process, rather than a single absolute value, which can more accurately reflect the treatment response; Second, based on CEA, a simple and dynamic efficacy prediction model is constructed, providing an auxiliary decision-making tool for clinicians, which helps to promptly adjust the treatment plan and promote individualized treatment; Third, the efficacy assessment is associated with DFS, providing comprehensive prognostic information from “whether it is effective” to “how long the benefit lasts”.

## 6 Limitations analysis

This study has certain limitations: Firstly, this study is a retrospective case analysis. Although strict inclusion and exclusion criteria were adopted, it still cannot completely avoid selection bias and information bias. Secondly, due to the limitations of follow-up time and the diversity of subsequent treatment plans, this study did not analyze OS, focusing only on DFS. Moreover, This study is a single-center data, although the sample size reached 257 cases, further verification by multi-center and large-sample studies is still needed. Additionally, this study did not include key variables that may affect prognosis, such as KRAS/NRAS/BRAF gene mutation status, microsatellite instability, and previous adjuvant treatment history. These factors may cause certain interference to the results. Only a small number of patients underwent ctDNA testing, so they were not included in this analysis.

## 7 Future prospects

Future research can be conducted in the following directions: carry out prospective, multi-center cohort studies to verify the predictive value of CEA dynamic changes for the efficacy of the CAPOX regimen; explore the optimal cutoff value of CEA changes (such as percentage decrease) and the prediction model combining other tumor markers (such as CA19-9, CA242) or inflammatory indicators (such as NLR, PLR); compare CEA dynamic changes with new biomarkers such as ctDNA, circulating tumor cells, etc., to evaluate their relative predictive efficacy; conduct research on adaptive treatment strategies based on CEA guidance, and evaluate whether early switching of the treatment plan can improve the OS of patients.

In addition to serum testing, since CEA can be secreted through the cell membrane and enter the extracellular space, as well as various body fluids such as peripheral body fluids and cerebrospinal fluid, it can also be detected in these fluids. According to the recommendations of the American Society of Clinical Oncology, CRC patients should have their CEA levels tested before initiating treatment, and this test should be repeated every 3 months for 2 consecutive years; then it should be changed to once every 6 months, with a total follow-up detection period of 5 years (22). However, some studies have shown that urine CEA testing is more meaningful than serum CEA testing (23). For instance, a study measured the levels of CEA in the urine and serum of 10 normal controls and 43 patients with bladder cancer (including 22 cases after schistosomiasis and 21 cases without schistosomiasis). The data on metastatic CRC show that the quantitative trajectory of ctDNA is more predictive than the qualitative results. Those with persistently high levels have the poorest survival (23.3 months), while those who have decreased levels from high to low (50.9 months) can approach the baseline low-level population (24). Therefore, in the future, a multi-dimensional nomogram integrating ctDNA abundance, CEA dynamics, and RECIST scores should be constructed.

In conclusion, the dynamic changes of serum CEA are independent predictors of the therapeutic efficacy and DFS of CRC patients receiving the CAPOX chemotherapy regimen. A decrease or stable normal level of CEA indicates a favorable prognosis, while an increase in CEA suggests an increased risk of disease progression. This indicator is worthy of being widely applied in clinical practice. Furthermore, in future studies, a prospective approach should be adopted, and CEA and ctDNA should be simultaneously and concurrently tested at the same time point to evaluate their efficacy (25).

## 8 Conclusion

This study conducted a retrospective analysis of the clinical data of 257 patients with CRC who received CAPOX chemotherapy. It systematically explored the relationship between the dynamic changes of serum CEA and the therapeutic efficacy and DFS of the patients. Based on the research results, it can be concluded that the dynamic changes of serum CEA during chemotherapy can serve as an effective predictive indicator for the therapeutic efficacy and prognosis of CRC patients using the CAPOX chemotherapy regimen. This predictive value is primarily significant in TNM Stage III patients. Patients with a decrease or stable normal level of CEA exhibit longer DFS and lower risks of disease progression, suggesting that this indicator has practical value in early identification of chemotherapy-sensitive populations and can provide more reliable decision support for individualized chemotherapy of CRC.

## Data Availability

The original contributions presented in the study are included in the article/supplementary material. Further inquiries can be directed to the corresponding authors.
